# Liquid biopsy for pediatric central nervous system tumors

**DOI:** 10.1038/s41698-018-0072-z

**Published:** 2018-12-17

**Authors:** Erin R. Bonner, Miriam Bornhorst, Roger J. Packer, Javad Nazarian

**Affiliations:** 1 Center for Genetic Medicine, Children’s National Health System, Washington, DC 20010 USA; 2 Institute for Biomedical Sciences, The George Washington University School of Medicine and Health Sciences, Washington, DC 20052 USA; 3 Brain Tumor Institute, Children’s National Health System, Washington, DC 20010 USA; 4 Department of Genomics and Precision Medicine, The George Washington University School of Medicine and Health Sciences, Washington, DC 20052 USA

## Abstract

Central nervous system (CNS) tumors are the most common solid tumors in children, and the leading cause of cancer-related death. Over the past decade, molecular profiling has been incorporated into treatment for pediatric CNS tumors, allowing for a more personalized approach to therapy. Through the identification of tumor-specific changes, it is now possible to diagnose, assign a prognostic subgroup, and develop targeted chemotherapeutic treatment plans for many cancer types. The successful incorporation of informative liquid biopsies, where the liquid biome is interrogated for tumor-associated molecular clues, has the potential to greatly complement the precision-based approach to treatment, and ultimately, to improve clinical outcomes for children with CNS tumors. In this article, the current application of liquid biopsy in cancer therapy will be reviewed, as will its potential for the diagnosis and therapeutic monitoring of pediatric CNS tumors.

## Introduction

Pediatric central nervous system (CNS) tumors are the leading cause of cancer-related death in children under the age of 19.^1^ Classically, these tumors were diagnosed by magnetic resonance imaging (MRI) and immunohistochemical assessment of surgically removed tissue. More recently, the World Health Organization (WHO) has begun incorporating molecular findings into the diagnosis of specific tumor types.^2^ Over time, it is likely that the detection and monitoring of molecular alterations will be critical for the clinical management of these tumors.^3^

While tumor resection for a subset of pediatric CNS tumors (e.g., frontal low-grade gliomas) can be both diagnostic and curative, many CNS tumors (e.g., diffuse midline gliomas) are not amenable to extensive surgical resection, either due to the infiltrating nature of the tumor or to its sensitive neuroanatomical location. For these tumors, stereotactic or open biopsy is often performed, but even these less invasive procedures carry the risk of serious surgical complications, and provide limited amounts of tumor tissue for pathologic and molecular diagnoses.^4,5^ Tissue biopsy is also subject to sampling bias, and tissue from a single tumor location may fail to capture intratumor heterogeneity.^6–9^ Longitudinal monitoring and assessment of tumor molecular events throughout the course of treatment also remains a challenge, since tumor resection or biopsy is often performed at diagnosis or recurrence, but not throughout the course of disease.

Current methods of monitoring pediatric CNS tumor response to therapy (MRI and clinical evaluation), are also limited in both sensitivity and specificity. For example, pseudoprogression (transient inflammation of the tumor region) resembles tumor progression on MR imaging, and may falsely inform treatment decisions.^10–13^ In addition, MR imaging cannot detect very small tumors and does not provide information about molecular changes that may be taking place within the tumor.

Pediatric CNS tumors demonstrate high need for minimally invasive molecular profiling of the tumor, and molecularly driven monitoring of tumor response and progression. Liquid biopsy, where the liquid biome is interrogated for detection of tumor-associated molecules, is a promising platform that can be used to address these limitations. In this review, liquid biopsy will first be described, and then its application and potential for providing next generation precision medicine for children diagnosed with CNS tumors will be discussed.

## Liquid biome

The “liquid biome” refers to biological fluids (biofluids) including blood, urine and cerebrospinal fluid (CSF). Biofluids may contain small amounts of tumor cells or biomolecules, including circulating tumor cells (CTCs), cell free DNA (cfDNA), circulating tumor DNA (ctDNA) and RNA (ctRNA), microRNAs (miRNAs), fragmented peptides, and intact proteins. The concept of the liquid biome for detection of circulating tumor molecules is not new. In fact, CTCs were reported in the blood of a deceased cancer patient as early as 1869,^14^ and in 1996, ctDNA was discovered in the plasma of lung cancer patients.^15^ Despite the scientific community’s awareness of tumor biomarkers in circulation, technologies necessary to exploit the full potential of the liquid biome in cancer management have not been available until recently.

For circulating biomarkers to be clinically useful, they must be highly tumor-specific and present in detectable concentrations. One example is CTCs, which detach from the solid tumor mass and flow through circulation.^16^ CTCs represent 1 in 10^9^ cells in peripheral blood,^16^ and are often detected through either positive or negative selection of specific tumor cell markers, which distinguish them from non-tumor cells.^16–19^ Because CTCs harbor tumor DNA, RNA, and proteins, they provide a rich source of information on the molecular biology of single tumor cells, and can shed light on mechanisms of clonal evolution, invasion and metastasis.^20^ Quantification of CTC abundance in peripheral blood may be used to monitor disease burden and progression, as shown in several recent publications.^16,21,22^ This is particularly useful for assessing a patient’s risk (i.e., identifying patients with metastasis), tumor recurrence, and response to therapy, which would be essential in the clinical management of children with CNS tumors.

cfDNA refers to fragments of DNA that are shed primarily by dying cells into biofluids.^23^ High levels of cfDNA in plasma of patients with advanced or progressive tumors has been associated with lower survival when compared to patients with lower cfDNA yield.^24,25^ Since cfDNA levels can be influenced by multiple factors, including increased white blood cell production, impaired renal function, and increased normal cell turnover, longitudinal monitoring of cfDNA levels will likely be more beneficial than a single time-point.^26^

The fraction of cfDNA that arises from tumor cells is called ctDNA.^27^ ctDNA are shorter and more fragmented than non-tumor DNA,^28^ and represent 0.01–10% of total cell free DNA in the blood, depending on tumor mitotic activity, treatment and tumor access to biofluids.^27^ ctDNA can be distinguished from background cfDNA based on the presence of tumor-specific mutations not found in the DNA of healthy cells, which provides a highly specific biomarker. ctDNA is thought to arise mainly from apoptotic and necrotic tumor cells, but may be released by any cell in the primary tumor and its metastatic lesions.^27,29^ As tumors grow, they undergo higher rates of cell turnover, releasing greater quantities of ctDNA.^29^ Thus, longitudinal monitoring of ctDNA abundance provides a traceable biomarker for assessing tumor burden. Given the relatively short half-life (~ 2 h) of ctDNA, real-time monitoring of tumor response by ctDNA profiling becomes a possibility.^27^ Indeed, highly sensitive assays have enabled detection of ctDNA harboring disease-specific mutations in the blood of patients diagnosed with colorectal,^30–32^ lung,^33,34^ and breast cancers,^35^ and in the CSF of adult^36,37^ and pediatric brain tumor patients.^38,39^ In these publications, ctDNA not only aids in diagnosis, but is also used to follow treatment response and progression. An advantage of ctDNA compared to CTC analysis is the relatively low amount of biofluid required to assess tumor mutation burden. For example, ongoing studies have shown that only 1 mL of plasma is sufficient for monitoring tumor response to treatment (further discussed below).^39^ This makes ctDNA longitudinal monitoring more feasible in pediatric patients, for whom it can be challenging to obtain large sample volumes.

ctRNA is also present in the liquid biome, and can be analyzed to inform potential fusion variants and changes in gene expression.^40,41^ This would be ideal for pediatric CNS tumors that are molecularly characterized by fusions, including KIAA1549-BRAF fusion positive low-grade gliomas, and by elevated transcript levels due to changes in gene expression or copy number alterations (CNAs), such as those occurring in medulloblastoma.^8,42^ However, due to vulnerability of single-stranded mRNA to degradation, ctRNA analysis is often more challenging than ctDNA.^43^

Unique, tumor-derived miRNA profiles have been identified in biofluids of adult glioblastoma, glioma, meningioma, adult medulloblastoma, and pediatric medulloblastoma.^44–47^ In adult glioma, strong downregulation of miR-122 in plasma is shown to be associated with disease progression and poorer prognosis.^48^ miRNA can also be used to detect patients with cancer and distinguish between cancer types. For example, high levels of miR-21 and miR-10b in the CSF of patients with glioblastoma or metastases from breast and lung cancer separates these patients from those who are either in remission or have non-neoplastic conditions, while miR-200a, miR-200b, miR-200c, and miR-141 are highly expressed in the CSF of patients with metastatic cancer but not primary brain tumors such as glioblastoma.^47^

Extracellular vesicles (EVs), including exosomes and microvesicles, also contain tumor-specific DNA, RNA and proteins, and therefore serve as a promising source of tumor biomarkers.^49^ Some studies have suggested that up to 93% of detectable cfDNA in plasma may be contained in exosomes, representing a potent source of tumor DNA for analysis.^49^ In serum of adult patients with glioblastoma, signature exosomal small non-coding RNA profiles have been identified and associated with glioblastoma diagnosis.^50^ miRNAs detected in plasma or serum are also often encapsulated into extracellular vesicles, protecting them from RNase digestion and enhancing their usefulness in determining diagnosis, prognosis, and therapeutic response in patients with CNS tumors.^51^ As such, overexpression of miR-21, mir-222 and miR-124-3p in serum exosomes has been shown to distinguish between high and low-grade gliomas, and to decrease post-surgical tumor resection, emphasizing the utility of EV-derived biomarkers as a means to determine tumor size and behavior.^52^ Proteins in extracellular vesicles can also provide information about tumor behavior. For example, high levels of proteins associated with cell invasion, including several proteins involved in regulation of invadopodia production, were found in patients with high-grade gliomas when compared to patients with low-grade gliomas.^53^

Proteins (intact proteins and peptide fragments) may also be directly secreted by tumor cells into the liquid biome. Proteins are stable and detectable in many types of biofluids, making them ideal biomarkers for the diagnosis and monitoring of many types of adult cancer.^54–59^ Proteins also show promise as biomarkers in pediatric CNS tumors. For example, studies have shown successful detection of tumor-associated proteins, including Cyclophillin A (CypA) and dimethylarginase 1 (DDAH1), in biofluids (urine, blood, and CSF) of children diagnosed with DIPG.^60^ Candidate biomarker proteins such as procollagen C-endopeptidase enhancer 1 (PCOLCE) have also been identified and linked to metastatic spread in CSF of children with CNS tumors,^61^ and enrichment of the protease inhibitor TIMP3 and growth factor bFGF in urine can help distinguish juvenile pilocytic astrocytomas from medulloblastoma and glioblastomas.^62^ Studying secreted peptides can also inform posttranslational modifications (PTMs), which may infer tumor response to treatment. Indeed, PTMs including lysine acetylation and arginine methylation of histone proteins have been detected in plasma and serum of patients with leukemia, breast and lung cancers.^63^

The tumor immunosignature is another potential biomarker that can be used for diagnosis and to inform targeted therapy for children with CNS tumors.^64^ During cancer development, the immune system produces antibodies against tumor-specific antigens that can be detected in the blood using methods such as high density peptide microarrays.^65,66^ Different adult cancer types have demonstrated unique immunosignatures that can be detected with high sensitivity and specificity in plasma or serum samples.^64^ Immunosignature profiles have been used in glioblastoma multiforme to detect tumor grade and *MGMT* promoter methylation status, which is predictive of response to temozolomide treatment in adults.^67^ Immunogenic profiling also provides the opportunity for targeted therapy, such as antigen-specific vaccines, that can adapt to changes within the tumor without requiring surgical resection.

These examples highlight several key nervous system tumor biomarkers present in the liquid biome, and strongly suggest the utility of these biomarkers in diagnosing, monitoring, and understanding tumor molecular alterations in various types of cancer. Although primarily for research, commercially available kits have been developed for biomarker isolation from biofluids including CSF, plasma, serum, and urine.^16,18,68–70^ These methods of isolation are continuing to evolve as more detailed characterizations of each biomarker comes to light, allowing for rapid, sensitive, and specific analysis of each biomarker, and greatly expanding the clinical potential of biomarkers in pediatric patients.

## Methods of analysis

Major sources of CNS tumor biomarkers are CSF, blood (plasma or serum), and urine. CSF is the most abundant source of biomarkers for CNS tumors, due to its close proximity to the tumor mass.^38,71^ Blood and urine have lower levels of ctDNA and other tumor biomarkers when compared to CSF,^72^ but this can be overcome through the use of highly sensitive platforms, suitable to detect and quantify low levels of tumor-derived molecules. When identifying a liquid source of biomarkers, it is important to find a balance between discomfort and risk to the patient, and obtaining sufficient levels of tumor biomarkers for analysis. Therefore, while CSF may serve as a better upfront medium for preliminary diagnosis and to detect commonly occurring mutations (using targeted sequencing and/or digital droplet PCR (ddPCR)), blood or urine may be a preferable source for long-term monitoring of variants associated with tumor response and progression.

The method of analysis will also be important to consider, depending on the liquid source. Highly sensitive methods are required for the detection and analysis of circulating tumor DNA, RNAs, and proteins within the liquid biome. Each biomarker is unique, requiring the presence of multiple different analytical methods, which can be adapted for different tumor types. For example, in some cases ctRNA is needed for detection of fusion variants that characterize a certain tumor type. In other instances, single gene mutations in ctDNA can be used to diagnose tumor subclass. Others may require detection of multiple mutation sites across the genome, or methods such as genome bisulfite sequencing or chromatin immunoprecipitation sequencing (ChIP-seq) to identify protein-DNA interactions or differential methylation patterns within CTCs.^73^ Different methods of analysis that can be applied to pediatric CNS tumors are highlighted below and in Tables 1 and 2.

**Table 1:** Examples of what has been accomplished using digital droplet PCR, alone or in combination with BEAMing PCR or custom amplicon sequencing, with a focus on CNS cancers

Method	Cancer type	Input and source	Biomarkers detected	Sensitivity, specificity	Reference
Digital droplet PCR	DIPG and other pediatric midline gliomas	10.5 ng DNA from CSF	*H3F3A* and *HIST1H3B* K27M mutation	87.5% sensitivity, 100% specificity	^38^
		150 bp DNA fragments via centrifugation			
	Glioblastoma	mRNA from extracellular vesicles in CSF (cisternal or LP)	*EGFR(v)III* amplification	61% sensitivity, 98% specificity	^41^
	High-grade glioma	mRNA transcripts from exosomes; 2–3 mL serum	*EGFR(v)III* amplification	81.58% sensitivity, 79.31% specificity	^40^
	High and low-grade gliomas	Cell free DNA from serum	Alu hypomethylation in high grade compared to low-grade gliomas		^121^
Digital droplet and BEAMing PCR	Adult glioma	mRNA isolated from extracellular vesicles in 1 mL CSF (cisterns and LP) and serum	*IDH1* G395A mutant transcripts		^92^
Digital droplet PCR and custom amplicon sequencing	Adult diffuse glioma	2–5 ng cell free DNA from 2 mL CSF, obtained from cisterna magna tap during autopsy and LP	*TERT* promoter mutations (C228T, C250T), *H3F3A* and *HIST1H3B* (K27M), *IDH1, IDH2, TP53* point mutations		^37^
			Targeted exon sequencing of *IDH1, IDH2, ATRX*, and *TP53*

**Table 2:** Examples of liquid biopsy applications, including analysis of CTCs, cell free DNA and protein biomarkers, with a focus on CNS cancers

Method	Cancer type	Input and source	Biomarkers detected	Sensitivity, specificity	Reference
Exome sequencing	Neuroblastoma	100 μL–3.3 mL plasma	*ALK* alterations	100x coverage	^94^
			MAPK pathway alterations		
			*MYCN* amplification		
			1p and 11q chr deletions		
			17q chr gain		
Targeted exon sequencing	Hepatocellular carcinoma	5-30 ng cell free DNA from 10 mL blood per patient	*MET, TP53, EGFR, PIK3CA, BRAF, ARID1A, PTEN, CDKN2A, CTNNB1*	15,000x average coverage depth, 0.1% limit of detection	^95^
Targeted panel sequencing	Non-small cell lung cancer with leptomeningeal metastases	CTCs from 7.5 mL peripheral blood, and 5–7.5 mL CSF	*MET* amplification, *ERBB2* and *EGFR* mutations	89.5% concordance of CTC DNA to matched tumor. 95.2% sensitivity for detecting metastasis	^96^
Shotgun massively parallel bisulfite sequencing	Neuroendocrine tumor, hepatocellular and smooth muscle carcinomas, nasopharyngeal, breast, and lung cancers	cfDNA extracted from 4 mL plasma	Detecting global hypomethylation of cfDNA genome, and copy number alterations	68% sensitivity, 94% specificity at 10 million reads per sample	^97^
CAPP-Seq	Lung cancer, lymphoma, leiomyosarcoma	cfDNA from 3–5 mL plasma	Indels, single nucleotide variants, rearrangements, copy number alterations	Variant detection as low as 0.01% frequency	^112, 122– 125^
		32 ng cfDNA for library prep (range 18.3–32 ng)			
LC-MS/MS, reverse phase protein array, Western blot, and ELISA	Pediatric CNS tumors including ATRT, ependymoma, malignant gliomas, medulloblastoma, PNET	Proteins isolated from CSF	Identification of candidate biomarkers predictive of metastasis		^61^

### Digital droplet PCR

ddPCR is capable of sensitively detecting and quantifying the allelic frequency of circulating tumor DNA fragments against a backdrop of non-tumor DNA.^74^ By using sequence-specific probes and primers that selectively bind to the mutant and wild type alleles of a target gene, ddPCR allows for the detection of very low frequency variants, and can provide an assessment of mutational burden. This is particularly useful for the serial monitoring of mutation allelic frequency (MAF) to track changes related to tumor clonal evolution, as well as tumor progression/regression (reflected in the abundance of mutant DNA detected). To maximize diagnostic utility of a single sample, these reactions can be multiplexed to detect up to four target sequences (two mutant, two wild type), using targeted primers and probes.^75^

Due to its ability to detect and quantify rare mutant alleles using very low levels of ctDNA, ddPCR has increasingly become the platform of choice in preclinical studies of colorectal,^76^ breast,^35^ melanoma,^77^ lymphoma,^78^ brain,^75,79^ and other cancer types^80^ (Table 1). In a recent publication, our group showed that ddPCR monitoring of single nucleotide variants associated with histone H3 (H3K27M mutation), both in blood and CSF, can be effective for tumor subtyping and following tumor response in DIPG and other midline tumors.^39^ In patients with Langerhans Cell Histiocytosis (LCH) and Erdheim-Chester Disease (ECD), *BRAF* V600E-positive cells can be detected in the urine and plasma, which has assisted with diagnosis and monitoring of patients treated with molecularly targeted therapy.^81,82^ Given the success of this method in LCH and ECD patients, this could potentially be expanded for other pediatric patients with *BRAF* V600E-positive tumors, such as gliomas (Table 3).

**Table 3:** Examples of key molecular alterations identified in the most common pediatric nervous system tumors, which could potentially be the focus of liquid biopsy-based mutation screening. Detection of these alterations in the liquid biome could facilitate diagnosis, classification of patients into molecular subgroups, qualification or disqualification from clinical trials, prediction of prognosis and detection of clonal evolution

Type of pediatric nervous system tumor	Molecular alterations	References
High grade glioma—hemispheric	*H3F3A* G34R/V with alterations in:	^119, 126, 127^
	* ATRX/DAXX*	
	* TP53*	
	* MYCN* amplification	
	* BRAF* V600E	
	* PDGFRA* amplification	
	*SETD2*	
	*IDH*	
	*BRAF V600E* +/− *CDKN2A/B*	
	*NTRK1, 2*, or *3* fusion (Infant HGG)	
High grade glioma—midline	*H3F3A* or *HIST1H3B* K27M with alterations in:	^119, 126^
	* TP53*	
	* ATRX/DAXX* (low frequency)	
	* FGFR1* (thalamic gliomas)	
	* NF1*	
	* PDGFRA* amplification	
	*BRAF* V600E +/− *CDKN2A/B*	
Atypical teratoid rhabdoid tumors (ATRT)	*SMARCB1*	^128^
	*SMARCA4* (inactivating mutations or deletions)	
Low-grade gliomas (brain or spinal cord)	*BRAF-KIAA* fusion	^42, 129, 130^
	*BRAFV600E*	
	*NF1*	
	*FGFR* fusions, *FGFR1* mutations, *NTRK* rearrangements	
Choroid plexus tumors	*TP53*	^131^
	Aneuploidy	
	Chromosome gains or losses	
Medulloblastoma	Shh pathway alterations	^8, 132^
	Wnt pathway alterations	
	*Myc, MYCN* amplification	
	*GLI2* amplification	
	*TP53*	
Ependymoma (papillary, clear cell, tanycytic, or anaplastic)	Supratentorial: *RELA* fusion,*YAP* fusion	^133^
	Infratentorial: PFA vs PFB based on methylation profiles	
	Spine/brain: *NF2*	
Embryonal tumors with multilayered rosettes (ETMR) and other embryonal tumors	C19MC-amplification (miRNA cluster), or fusion with TTYH1 gene	^129, 134^

In addition to single nucleotide variants, ddPCR can detect differentially methylated cfDNA, which has been particularly useful for colorectal cancer in adults.^83–86^ This has clear application to pediatric CNS cancers such as pediatric high grade gliomas, ependymomas, and embryonal tumors, which harbor relatively low mutation load but have distinct methylation patterns.^87,88^ As such, ddPCR provides a rapid, affordable, and sensitive method for detecting distinct CNS tumor markers in pediatric patients.

### BEAMing PCR

Another highly sensitive platform for detecting tumor mutations in cfDNA is beads, emulsification, amplification and magnetics PCR (BEAMing PCR). In BEAMing PCR, individual DNA sequences are amplified, fluorescently-labeled, and quantified using flow cytometry. Like ddPCR, BEAMing assays can be multiplexed for identification of multiple mutations in a single sample, maximizing diagnostic utility and requiring fewer samples for analysis.^89^ These PCR-based approaches are highly sensitive and detect tumor-specific variants at a frequency as low as 0.001%, provide a measurement of mutation load, and enable identification of residual disease post-treatment.^90^ BEAMing PCR has shown promise in detecting mutations in preclinical studies of adult colorectal,^27,30–32^ lung,^33^ breast,^91^ and glioma^92^ (Table 1). Similar to ddPCR, BEAMing PCR has fast turn-around time and is more cost effective compared to next generation sequencing, making this an attractive method for real-time diagnosis and therapeutic monitoring for children with CNS tumors.

### Next generation sequencing

Although PCR-based methods are sufficient at detecting mutations in very low levels of ctDNA, they are limited by the number of mutations that can be detected, and perform best when targeting a single nucleotide variant. However, multiple genes may be mutated in pediatric CNS cancers, each with multiple alterations rather than a unique nucleotide change (Table 3).^93^ Detecting mutations in these genes using a PCR-based approach would require dozens of probes and primers, which is both costly and inefficient. Next generation sequencing (NGS) of ctDNA allows for the detection of a wide range of alterations, including unknown variants, typically through targeted exon sequencing. This approach has been used in neuroblastoma, and in adult cancers including glioblastoma, gastrointestinal, genitourinary, breast, and lung cancers.^36,94–96^ NGS would be ideal for the discovery of mutations acquired at the time of progression, or the detection of mutations in ctDNA from patients where the molecular profile of the tumor is unknown.

Compared to PCR, NGS is more expensive and time-consuming, and less sensitive, detecting variants as low as 0.1–10% frequency, compared to 0.001% in ddPCR and BEAMing PCR.^90^ In contrast to tissue-derived DNA, ctDNA is highly fragmented and short, and requires higher sequencing depth. To accommodate this, new NGS platforms that amplify and sequence specific regions of the genome are being developed and optimized for use with low input, fragmented ctDNA (Table 2). Thus, NGS technology has the potential to revolutionize the approach to diagnosis and clinical management of pediatric CNS cancers.

### Alternative technologies

Massively parallel bisulfite sequencing is being used to detect genome-wide methylation changes in plasma cfDNA. In this method, unmethylated cytosine is converted to uracil, and uracil is then converted to thymine during PCR amplification, allowing methylated cytosines to be identified.^97^ This has been used to detect hypomethylation in cfDNA in plasma of cancer patients with high sensitivity and specificity.^97^ For example, in esophageal cancers, genome-wide DNA methylation profiling of serum cfDNA has shown the methylation profile of cfDNA to be highly concordant with tumor tissue DNA.^98^ As noted previously, this method would be beneficial for detecting and monitoring levels of ctDNA marked by distinct methylation patterns in children with CNS tumors.

For cell free RNA, miRNA and exosomal RNA isolation, whole blood is collected in tubes containing RNA-stabilizing preservatives.^40,41,99^ RNA can then be reverse transcribed to complementary DNA, and used to detect fusion variants via next generation sequencing or ddPCR of cDNA. This has been useful for the detection of *EGFR(v)III* amplification in high-grade gliomas, and *IDH1* G395A mutant transcripts in adult glioma (Table 1).^40,41,92^ Some pediatric CNS tumors, such as gliomas and ependymomas, are primarily characterized by fusion variants (i.e., KIAA1567-BRAF, RELA, NTRK fusions (Table 3)). For these tumors, RNA would likely be more informative as a biomarker than ctDNA.

Liquid chromatography-mass spectrometry (LC-MS) is a commonly used method for protein biomarker discovery and quantification (including quantification of tumor-specific missense mutant proteins) in specimens including plasma, serum, urine and tissue.^61,100^ LC-MS methods are being developed to minimize protein loss and degradation, and to achieve deep proteome profiling in plasma.^101^ LC-MS following immunoaffinity purification has been used to identify tumor HLA antigens in plasma of adults with glioblastoma.^102^ In addition to high resolution mass spectrometry, methods such as ELISAs (enzyme linked immunosorbent assays) can quantify protein levels in blood. For example, programmed cell death ligand-1 (PD-L1) has been quantified in pre-treatment serum of patients with hepatocellular carcinoma, and shown to be predictive of disease-free and overall survival.^103^ Additional approaches to protein analysis use a top-down approach, in which the entire proteoform (the diversity of forms that can arise from a single protein-coding gene, including those produced by posttranslational modifications and alternative splicing) is analyzed in its undigested form.^104^ This approach was used to analyze intact proteins in the CSF of pediatric patients with posterior cranial fossa brain tumors, leading to identification of LVV- and VV-hemorphin-7 as potential biomarkers for this tumor type.^105^ Despite the potential, there remains a need to develop more robust bioinformatics pipelines for proteomic analyses,^106^ particularly for complex proteoforms isolated through the top-down approach.^107^ With further optimization, quantification and longitudinal monitoring of protein biomarkers have clear application to pediatric CNS tumors.

## Liquid biopsy for pediatric CNS tumors: challenges and future directions

There are several key challenges in the implementation of liquid biopsy for the clinical care of pediatric CNS tumor patients. These platforms are not certified by CLIA (Clinical Laboratory Improvement Amendments) regulations, and there are a limited number of facilities that have the training and tools required for this type of liquid biopsy analysis. Additionally, methods of collecting and processing liquid biopsy specimens have not been standardized and optimized for downstream analysis of each biomarker type. For example, ctDNA integrity is affected by factors such as temperature and storage conditions,^108,109^ and time-dependent increases in cfDNA quantity have been observed in blood samples due to leukocyte lysis, which can increase total levels of cfDNA and dilute the fraction of tumor-specific DNA below the level of detection.^110^ In addition to the pre-analytic conditions such as collection, processing, and storage, other variables such as patient gender, age, lifestyle, and exposure to medications may also affect protein analysis, and should be considered during interpretation of the results.^106^ Therefore, before liquid biopsy can become a standard-of-care test, collection and preparation conditions for different biofluids and biomarker types will need to be standardized across institutions.

Another challenge is the sample volume required by many assays currently used to analyze liquid biopsy samples. Due to the low levels of tumor biomarkers in the liquid biome, in order to achieve a sufficient quantity of CTCs, ctDNA, ctRNA and other biomarkers, high sample volumes may be necessary. For example, up to 10 mL of blood and 7.5 mL of CSF have been used for biomarker discovery in adult patients (Table 2), which can be difficult to obtain in young children. High sensitivity assays, designed to detect and amplify low levels of tumor biomarkers, can be used in the pediatric population to overcome this challenge. For example, using ddPCR for commonly occuring mutations, or the top-down approach for protein isolation, can improve biomarker detection and recovery, allowing for the collection of smaller sample volumes.^38,104^ Careful selection of the liquid source can also decrease volume requirements in pediatric patients. Since tumor biomarker concentrations are highest in close proximity to the tumor,^71^ CSF may be the preferred liquid source for biomarkers that are present in low concentrations, such as ctDNA and ctRNA, while blood and urine could be used for CTCs, EVs, or protein biomarkers.

As mentioned above, sequencing fragmented ctDNA also poses a challenge. The short size of the DNA fragments makes sequencing more difficult, and individual read lengths can be subjected to adapter contamination, resulting in misalignment of the DNA molecule to the reference genome or a low alignment score, causing the read to be discarded.^111^ Because of this, low frequency variants could be missed due to their small size and incompatibility with current sequencing methods intended for larger DNA fragments. cfDNA requires extremely sensitive, high-throughput sequencing platforms that reduce background noise, detect low frequency variants, and encompass the diversity of tumor molecular alterations.^111^ Cancer Personalized Profiling by Deep Sequencing (CAPP-Seq, Table 2) is an example of a new sequencing platform that optimizes library preparation methods for low DNA input, allowing for increased sensitivity despite low fluid volumes.^112^

Disruption of epigenetic mechanisms that affect DNA methylation, nucleosome positioning, long non-coding RNA and miRNA profiles, and chromatin accessibility, are also known to play a major role in early tumor development for pediatric CNS tumors such as medulloblastoma and gliomas.^113,114^ As noted previously, methods such as ChIP-seq and genome bisulfite sequencing are already being used to identify protein-DNA interactions or differential methylation patterns within CTCs of adult cancer patients,^73^ and could potentially be applied to the pediatric population. Assay for Transposase Accessible Chromatin with high-throughput sequencing (ATAC-seq), micrococcal nuclease-assisted isolation of nucleosomes sequencing (MNase-seq) and DNase I hypersensitive sites sequencing (DNase-seq) are being developed for adult tumor tissue to detect chromatin accessibility, particularly at regulatory sites such as promoters, enhancers, and insulators.^115–117^ Although not currently being used in the context of liquid biopsy, these novel approaches could potentially allow for rapid detection of epigenetic biomarkers in CTCs, and should be considered in pediatric CNS tumor biomarker research.

While each biomarker may play an independent role in tumor management, it is likely that biomarker combinations, such as protein plus ctDNA as seen in pancreatic cancer,^118^ may be even more useful for the early detection and monitoring of pediatric CNS tumors. Further studies that examine the role of each biomarker, both alone and in combination with other biomarkers, will be required to fully recognize the potential of liquid biopsy in children with CNS tumors. Finally, in order to capture the variety of alterations in pediatric CNS tumors (Table 3), distinct and specialized methods will need to be developed for accurate analysis of different types of alterations, including single nucleotide variants, fusions, copy number alterations, differential methylation patterns and miRNA profiles. The relatively small numbers of patients, and the limited number of institutions prioritizing pediatric CNS tumor biomarker research, pose a challenge in the discovery and technical validation of liquid biopsy platforms for this patient population. Multi-institutional collaborations will be important for combining liquid biopsy data (including data from CTC, ctDNA, ctRNA, exosome and protein analyses) together with clinical data, to more tightly define the predictive value of liquid-based biomarkers, and to correlate this data to clinical outcome—with a specific focus on the pediatric population.

## Conclusion

Over the past ten years, knowledge of common molecular alterations in pediatric central nervous system tumors has greatly expanded (Table 3). This data has come largely from sequencing of tumor tissue DNA, and has led to classification of tumors into molecular subgroups with distinct clinical outcomes,^8,119,120^ empowering development of therapeutics that target tumor subtype-specific alterations with greater precision. With increased understanding of the genomic landscape of pediatric CNS tumors, liquid biopsy will serve as a powerful tool to complement our current methods of diagnosis and management.

When used alongside conventional diagnostic methods, liquid biopsy can fill in information gaps that are otherwise not available. At diagnosis, liquid biopsy has the potential to (1) provide information about prognosis in conjunction with tumor histology and MRI characteristics; (2) supplement tumor biopsy by sampling multiple tumor cells, revealing mutations that may not be detected in biopsy tissue due to tumor heterogeneity; and/or (3) provide a diagnosis for patients who were not able to undergo biopsy, but have specific diagnostic alterations (such as an H3K27M mutation in a patient with a midline tumor) noted in liquid samples. During the treatment course, biomarker levels can be used alongside MRI to assess treatment response and help differentiate between tumor pseudoprogression and true progression. At progression, molecular profiling of ctDNA can detect new mutations, possibly before changes in the tumor are seen on MRI, allowing for earlier intervention and overall improved outcomes. Finally, molecular and epigenetic changes that occur in the tumor at progression can be identified in liquid samples, offering critical information that can be used to develop a new treatment plan without requiring a tissue biopsy.

Despite the challenges facing the implementation of liquid biopsy as a clinical tool for pediatric CNS tumor diagnosis and surveillance, these challenges can be overcome with continuing optimization of analytical methods, and with increases in sensitivity and specificity. Once clinically validated, the successful utilization of liquid biopsy in pediatric CNS tumors will address unmet needs in pediatric neuro-oncology, and will ultimately improve outcomes for these patients.

## Data Availability

No datasets were generated or analyzed during the current study.
